# Cellular and Molecular Preconditions for Retinal Pigment Epithelium (RPE) Natural Reprogramming during Retinal Regeneration in Urodela

**DOI:** 10.3390/biomedicines4040028

**Published:** 2016-12-01

**Authors:** Eleonora N. Grigoryan, Yuliya V. Markitantova

**Affiliations:** Kol’tsov Institute of Developmental Biology, Russian Academy of Sciences, Moscow 119334, Russia; yuliya.mark@gmail.com

**Keywords:** eye, retinal regeneration, retinal pigment epithelium, reprogramming, molecular prerequisites

## Abstract

Many regeneration processes in animals are based on the phenomenon of cell reprogramming followed by proliferation and differentiation in a different specialization direction. An insight into what makes natural (in vivo) cell reprogramming possible can help to solve a number of biomedical problems. In particular, the first problem is to reveal the intrinsic properties of the cells that are necessary and sufficient for reprogramming; the second, to evaluate these properties and, on this basis, to reveal potential endogenous sources for cell substitution in damaged tissues; and the third, to use the acquired data for developing approaches to in vitro cell reprogramming in order to obtain a cell reserve for damaged tissue repair. Normal cells of the retinal pigment epithelium (RPE) in newts (Urodela) can change their specialization and transform into retinal neurons and ganglion cells (i.e., actualize their retinogenic potential). Therefore, they can serve as a model that provides the possibility to identify factors of the initial competence of vertebrate cells for reprogramming in vivo. This review deals mainly with the endogenous properties of native newt RPE cells themselves and, to a lesser extent, with exogenous mechanisms regulating the process of reprogramming, which are actively discussed.

## 1. Introduction

The retinal pigmented epithelium (RPE) of the eye of adult vertebrates and human is represented by neuroectoderm-derived cells, packed into the monolayer located between neural retina (NR) and vascular choroidal coat (choroid). The RPE is composed of pigmented, polarized, hexagonal and highly specialized cells. Among the variety of well-known functions of this tissue, the most important is its participation in visual cycle, phagocytosis and digestion of photoreceptor disks, production and release of growth factors which regulate choroid–NR interconnections and behavior of RPE itself. It is also worth noting the barrier and transport functions of RPE and its participation in NR pattern formation in development. Being the main participant of NR sensory function, RPE is a main target for the destructive effect of different endo- and exogenous factors leading to RPE pathology. The latter, in turn, is a cause of a severe retinal diseases like as retinal detachment and proliferative vitreoretinopathy (PVR) in human [1,2]. However, there are animals, such as some amphibians, that never demonstrate RPE pathology but, in contrast, are capable of NR regeneration in response to NR trauma. At the basis of this unique ability is the conversion of RPE cell type to neurons and glial cells via a transient population of RPE-derived neuroblasts.

## 2. Regeneration of Neural Retina (NR) from Retinal Pigmented Epithelium (RPE) in Urodela

Retinal regeneration from RPE cells is a long-known phenomenon that has been repeatedly described at different levels, from morphological to molecular genetic [3,4,5,6,7]. This process in the newt is initiated by detachment of photoreceptor outer segments from the apical RPE cell processes [8]. The tension of the RPE layer decreases, its attachment to the underlying Bruch’s membrane is weakened and eventually lost, and some cells come loose. Thus, the RPE partially breaks down but does not disintegrate, and its cell population recovers due to cell proliferation and redifferentiation during NR regeneration. In the course of NR regeneration’s start and progression, RPE cells undergo reprogramming, i.e., their initial differentiation as pigmented epithelial cells changes into that of retinal cells. This phenomenon, termed *transdifferentiation* [9], is consistently reproduced after any kind of split-up between NR and RPE, including detachment of the retina, its surgical removal, and degradation caused by cutting of the optic nerve and blood vessels [10]. Reprogramming involves the stage of active proliferation whereby RPE cells dedifferentiate, divide and give rise to an intermediate population of multipotent blast cells. This process is controlled by overlapping regulatory gene networks in which a special role is played by signal proteins and transcription factors [7,11,12,13,14].

**Figure 1:**
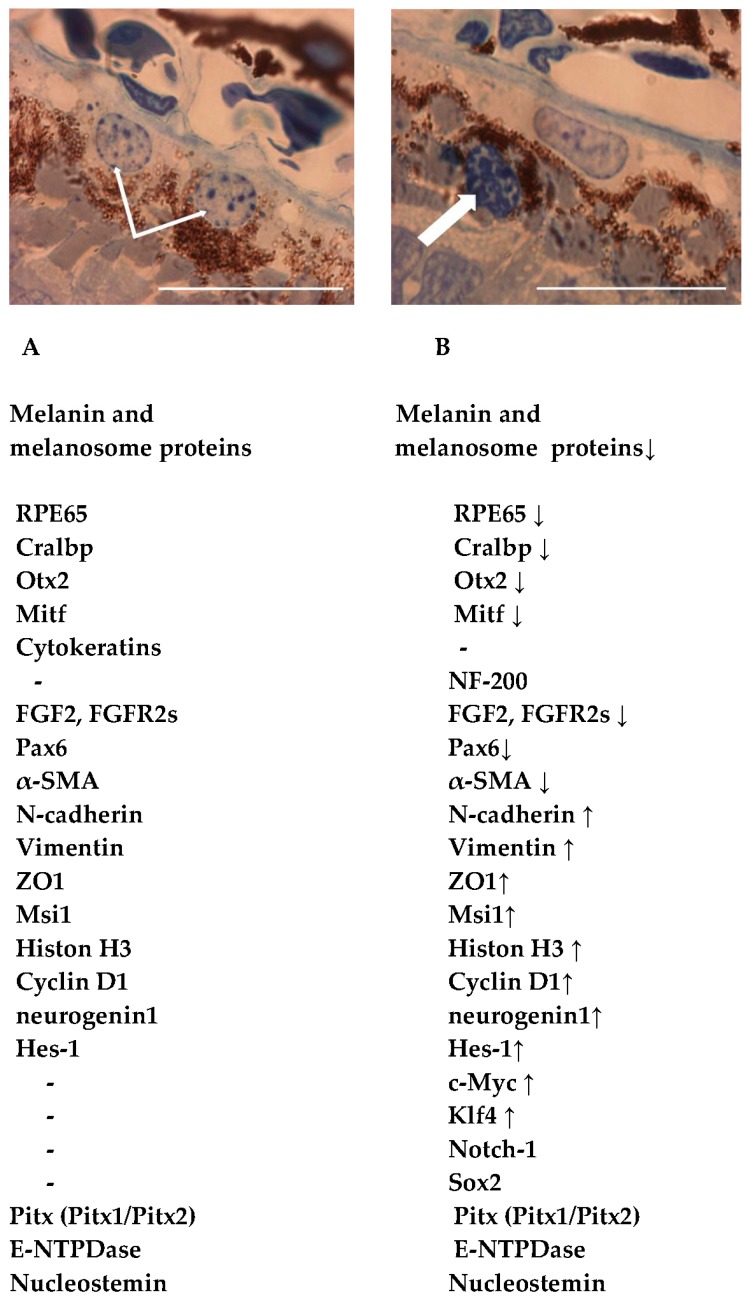
Accumulated data on morphological and molecular features of native retinal pigment epithelium (RPE) cells and those at the beginning of natural reprogramming to neuronal and glial cells of regenerating retina [6,7,11,12,13,14,17,18,19,20,21,22,23,24]. (**A**) RPE cells (thin white arrows) in the RPE layer of the newt *Pleurodeles waltl*; (**B**) RPE cell that left its layer and stays at the beginning of reprogramming (thick white arrow); Scale bar: 100 µm. See details in the text. Down- (**↓**) and up- (**↑**) regulation of gene/protein expression.

Evidently, amphibian RPE cells have competence for in vivo reprogramming into NR, which is rooted in the origin of both tissues from the same anlage, the posterior wall of the developing eye cup [15,16]. This competence, which underlies the RPE retinogenic potential, needs characterization in terms of cell and molecular biology. This review summarizes the results of our studies and relevant published data concerning mainly the endogenous properties of newt RPE cells that, as will be shown below, are conducive to their reprogramming and, eventually, epimorphic regeneration of the retina (Figure 1).

## 3. Newt Eye Development and Retention of Pedomorphic Features in the Retina

A good help in studies on RPE natural reprogramming in vivo is that ample information is available on the role of various molecules regulating the processes of vertebrate eye development during embryogenesis. The development of eye tissues in representatives of different taxa is under control of highly conserved signal molecules (e.g., Fgf, Tgf, Wnt, Shh, Notch, activin) and transcription factors (Pax6, Sox2, Six3, Rx1, Chx10, Optx2, Mitf, etc.) that specifically interact with DNA or protein factors [25]. Each of the above transcription factors can induce ectopic eye development, which is evidence for their key role in the functioning of regulatory gene networks [26].

The basic stages of eye development in the newt are the same as in other vertebrates, and the expression pattern of the main transcription factors is also similar among them. Thus, the multifunctional transcription factor encoded by the *Pax6* master gene and expressed during newt eye development is similar to that described in other species [6]. It is localized in both neuroblastic layers of the eye cup, of which one develops into the RPE and the other into the NR. The Pax6 protein is redistributed in the course of tissue specialization so that at later stages it marks differentiating neurons (ganglion, amacrine, and photoreceptor cells) in the NR, while in the RPE it is detected at low level. Thus, Pax6 differential expression at later stages of NR and RPE differentiation depends on the molecular context, the presence of specific binding sites, and signals from the cellular microenvironment [27].

The development of the newt eye has certain traits related to the phenomenon of pedomorphosis [28]. An important pedomorphic feature in the developing and definitive newt retina is that it contains “underdifferentiated” displaced bipolar cells with Landolt’s club. Upon retinal detachment, they move from the inner nuclear layer to the photoreceptor layer and differentiate into rods with an outer segment [29,30]. In addition, the retina of adult newts contains the zone of persistent slow growth, which includes the nonpigmented inner layer of the ciliary zone of the iris inner layer and the extreme peripheral area of the neural retina (*pars ciliaris + ora serrata)* [31]. The cells of this zone are morphologically undifferentiated and express genes and proteins that are markers of the eye field during early development [17]. Thus, the adult newt retains certain juvenile features with respect to the level of tissue differentiation. Specific features in its development have also been revealed at the molecular genetic level. In particular, it has been found that the hematological- and neurological-expressed sequence 1 protein (HN1), the product of the *Hn1* gene, is specifically expressed in an immature retina, with its subcellular localization changing (from nuclear to cytosolic) during retinogenesis [32]. The expression of the HN1 protein is highly activated at early stages of retinal formation in the newt but is not upregulated in mouse RPE cells that lack the ability to dedifferentiate into the progenitor cells. Therefore, the upregulation of the *Hn1* gene can serve as a marker for detecting dedifferentiated cells capable of reprogramming. Specific features are also observed in the expression of other genes responsible for specification and maintenance of low-differentiated cell status in the newt eye, particularly in the RPE (see below).

## 4. Differentiation of Newt RPE Cells and Conditions of Its Stabilization

Addressing the question of prerequisites for natural cell reprogramming, it is necessary to consider factors responsible for the stability of differentiation, since it is these factors that can allow or forbid the cells to enter the path of conversion. Melanogenesis is one of these factors. The presence of melanin granules in the cytoplasm is a natural specific marker of RPE cells. Our previous experiments with ^3^H-DOPA, the precursor of melanin synthesis, have shown that the normal RPE of adult newts retains the ability to synthesize and accumulate the pigment and that melanin-synthesizing cells concentrate at the RPE periphery, i.e., in the zone where cell proliferation is observed at the same time [33]. There is also experimental evidence for the loss/resynthesis of pigment granules in adult RPE cells of mammals and humans [34], but no relationship of pigmentation level and expression of melanogenesis-associated proteins with RPE transdifferentiation has been revealed in these studies. In contrast, the inhibition of melanin synthesis in newts is a factor of RPE cell reprogramming, which takes place against the background of increased proliferative activity [10,33]. It is also noteworthy that the loss of pigment after the cessation of its synthesis in newts occurs in two ways: on the one hand, fragments of the cytoplasm filled with pigment granules are phagocytized by macrophages (including RPE-derived “melanophages”); on the other hand, the amount of pigment granules is reduced in the course of cell divisions [35]. As a result, the pigment is rapidly eliminated, and the loss of this specific trait obviously facilitates the process of RPE cell dedifferentiation and reprogramming.

In addition to melanin, the process of RPE cell conversion involves changes in the expression of another specific protein, RPE65, which has isomerohydrolase activity and is responsible for 11-*cis*-retinal regeneration in the visual cycle [36]. Experiments with antibodies recognizing the newt homolog of RPE65 have shown that this protein in the *Сynops pyrrhogaster* eye is confined only to RPE cells [18]. Moreover, this protein has been detected not only in the normal RPE but also in RPE-derived dedifferentiated cells (neuroblasts) forming the cell rows of the regenerating NR’s early rudiment. These data are interpreted in different ways: as evidence that the initial characteristics of RPE cells during reprogramming may be combined with new, proneural characteristics or that the RPE65 protein detected in the RPE-derived retinal rudiment is the remainder after its incomplete degradation rather than newly synthesized protein. The second variant is more probable, since the expression of the gene encoding RPE65 has proved to be sharply downregulated at the early stage of reprogramming [18]. This conclusion is confirmed by our data on the suppression of the *Rpe65* gene in the course of retinal regeneration from the RPE of another newt species, *Pleurodeles waltl* [37]. The detachment of RPE from NR in mammals (rodents) also leads to the suppression of *Rpe65* expression, with the reduction in the amount of its transcripts coinciding with downregulation of genes coding for other proteins of the visual cycle [38]. Thus, the data on *Rpe65* expression are not indicative of any specific features that distinguish the RPE of newts from that of other vertebrates and are associated with its ability to transdifferentiate into neural cells. However, the detection of the RPE65 protein in depigmenting cells of the newt RPE, which have already entered the path of dedifferentiation, suggests the possibility of temporary “overlap” between their initial and new specific characteristics in the course of reprogramming. The main contribution to the determination and maintenance of RPE-specific cell differentiation is made by signal molecules and transcription factors. The range of relevant factors identified to date is relatively small. Thus, it has been shown that Wnt/beta-catenin signaling targeted at transcription factors such as Pax6, Mitf, and Otx2 plays an essential role in the development of RPE and the maintenance of its differentiation in lower and higher vertebrates [39,40,41,42]. Among several isoforms of the Mitf protein, the main role in the regulation of RPE melanogenic differentiation is played by the D-Mitf isoform [40]. The expression of the *Mitf* gene and its isoforms in the normal RPE of adult newts has not yet been sufficiently analyzed because of technical difficulties. Studies on another model—explanted chick embryo optic vesicles—has demonstrated the role of Wnt/beta-catenin signaling and activation of Otx2 transcription factor in inducing the expression of RPE-specific genes. It has been shown that D-Mitf activation can result from both the binding of beta-catenin and Otx2 with the *D-Mitf* gene enhancer and from autoregulation of the *Mitf-D* and *Otx2* gene expression [43].

Considering the behavior of higher vertebrate RPE cells in vitro, it should be noted that the stability of the epithelial RPE phenotype, cadherin-dependent cell adhesion, and the presence of melanin in the cells are in any way related to Wnt/beta-catenin signaling [44]. It is also known that inactivation of the Wnt/beta-catenin pathway in mouse embryos results in a decrease in the expression of RPE-specific transcription factors Mitf and Otx2 followed by the onset of expression developing NR markers Rx and Chx10 [39,43]. The function of the Wnt signal pathway in the normal newt RPE and during retinal regeneration has not yet been studied in detail. However, these data are relevant to the subject matter, because Wnt signaling has been shown to be an important component of reprogramming in experiments on induction of mouse embryonic fibroblasts into pluripotent stem cells [45].

In a recent study, an attempt has been made to identify molecules that block the regenerative activity of pigmented cells in the ciliary zone of the vertebrate eye [46]. The results show that a likely candidate is ephrin-A3 (the ephrins and EPH-related receptors comprise the largest subfamily of receptor protein-tyrosine kinases). This protein is upregulated in the early postnatal period, thereby creating a “strong prohibitory niche” for the above cells. This occurs due to activation of the EphA4 receptor and suppression of Wnt3a/β-catenin signaling. As a result, not only proliferation of these cells is inhibited, but also their morphology and specialization are firmly established. In other words, the intrinsic ability of pigmented ciliary body cells to produce different types of retinal neurons [47,48] is, in this case, abolished by negative regulators of cell proliferation and neuronal differentiation. It may well be that a similar mechanism is responsible for differences in the RPE retinogenic potential between the newt and mammals.

Stabilization of RPE differentiation in normal development is dependent on the expression of the *Otx2* gene, which, in turn, suppresses the expression of factors Sox2 and Fgf8 responsible for NR differentiation [49,50]. The pattern of *Otx2* expression in the RPE has also been studied in the course of cell conversion during NR regeneration in newts *C. pyrrhogaster* [19] and *Pl. waltl* [37]. It has been found that its expression is maintained in the intact RPE and at the onset of its reprogramming into the NR, against the background of cell entry to the phase of DNA synthesis and retention of RPE protein markers. As shown by in situ hybridization, *Otx2-*expressing cells are evenly distributed all over the RPE layer, both in its central zone (involved in NR regeneration) and the peripheral zone (not involved in it). There is evidence that *Otx2* is expressed for 14 days after the initiation of regeneration (NR removal) in approximately half of the RPE cell population, including cells expressing the PCNA proliferation marker [19]. Thus, as in the case of the RPE65 protein, the *Otx2* expression in RPE cells is apparently not an obstacle to their entry onto the path of reprogramming. Therefore, a distinctive feature of the newt RPE is that tissue-specific differentiation molecules are still expressed in it after the onset of reprogramming. Moreover, it appears that the expression of *Otx2* may even contribute to the process of reprogramming. Such a possibility follows from the data on *Otx2* reactivation (together with *RX1* and *SIX3*) by OCT4 and SOX2 during the generation of RPE cells from human iPSCs [51].

The group of regulatory molecules responsible for RPE specification during development also includes factors such as Bmp, Shh, and activin [52,53,54,55], but information on their expression in the newt RPE is far from being sufficient.

## 5. Proliferative Activity of Adult Newt RPE Cells in the Course of In Vivo Reprogramming

### 5.1. Persistent Cell Proliferation in the RPE

Proliferative activity is essential for the conversion of RPE cells into the NR in adult newts. Its pattern in the normal RPE and during its conversion into the NR in different newt species (*T. vulgaris*, *T. cristatus*, and *Pl. waltl*) has been studied by a number of methods in complete series of eye sections. By means of ^3^Н-thymidine pulse labeling, up to 3% of DNA-synthesizing cells and very few mitoses were revealed in the normal RPE of adult *T. vulgaris* newts [33]. The presence of such cells was subsequently confirmed in experiments on *Pl. waltl* newts using an original method of long-term delivery of BrdU (thymidine analog) and repeated ^3^Н-thymidine injections [56]. In all cases, the proliferative activity of normal RPE cells was characterized by a long S-phase, with mitoses being very rare. It should be noted that persistent, low-level proliferative activity is not unique to the adult newt RPE but has also been observed in the marginal zone of RPE in freshly hatched chicks [57] and adult rodents [58]. Rare BrdU-labeled cells at the periphery of the RPE were also revealed in our experiments with in vitro cultures of the posterior eye segments from adult albino rat [59].

The ability of amphibian RPE cells to pass from quiescence to active proliferation is conducive to their reprogramming. This process is initiated during the first week after the loss of contact with the NR [10]. After the cells begin to proliferate, they cease to synthesize melanin and gradually lose melanin granules (their basic trait), which are “diluted” as a result of cell divisions [10,33]. The duration of the cell cycle decreases during the proliferative stage of RPE reprogramming, and its parameters change: the G_1_ phase is reduced, while the S phase is prolonged [4]. It is also noteworthy that six to seven cell divisions of newt RPE cells are necessary for them to express the first signs of new specialization acquired in the course of in vivo reprogramming [60].

Thus, the low-level proliferative activity of the adult RPE is an intrinsic property of this tissue that is common to all vertebrates studied in this respect, but it is only in amphibians that this property is implemented for in vivo reprogramming and enlargement of the transitory cell population that subsequently differentiates into neurons and glial cells of the regenerating NR. Therefore, measures (treatments) aimed solely at stimulating in situ proliferative activity of RPE in mammals or humans are unlikely to results in the enhancement of its retinogenic potential. This conclusion is indirectly confirmed by data on the nonpigmented RPE of albino rats, which has ten times higher proliferative activity, compared to that of wild-type rats, but is incapable of changing its phenotype in vivo [58].

### 5.2. Mechanism of Activation of Newt RPE Cell Entry into the S-Phase

In a recent study aimed to determine the key molecular mechanism underlying natural reprogramming and regeneration in lower vertebrates [61] have devoted special attention to extracellular signal-regulated kinase (ERK) activation. They have shown that sustained ERK activation by serum in postmitotic salamander muscle cells results in their cell cycle re-entry that possibly linked with p53 downregulation. In parallel, ERK activity leads to epigenetic modifications and *Sox6* downregulation. The latter is one of the muscle-specific genes regarded as an aspect of dedifferentiation. It is important to note also that ERK activation is long term in salamander myotubes, while it is short and transient in myotubes of mammals. Regeneration-incompetent mammalian cells are incapable of inducing sustained ERK activation, most likely due to a lack of an upstream receptor or signaling component, and are therefore unable to undergo reprogramming [61].

The newt RPE has also been studied with respect to the molecular mechanism of cell entry into the S-phase after RPE separation from the NR [62,63]. The authors of the first study analyzed an activity of MEK-ERK signaling in RPE just after NR removal from the newt eye and found it activated within 30 min after retinectomy. In the second study, changes in the MEK–ERK cascade were analyzed in short-term in vitro cultures of the posterior eye segments without the NR, and activation of MEK–ERK was observed within 1 h after dissection. In both cases, the authors attribute these results to upregulation of each component of the MEK–ERK pathway by a positive feedback mechanism [62,63]. It is noteworthy that the timing of RPE cell entry into the phase of DNA synthesis differs depending on the newt species and the kind of surgery, but, in any case, this occurs within the first 10 days after RPE separation from the NR [10]. It remains to be found what occurs in the as-yet morphologically unchanged RPE layer during this period. Nevertheless, the results obtained show that RPE of the newt has the mechanism of signaling cascade fast start or its sustained activation, with consequent entry into the cell cycle and, hence, the process of reprogramming.

## 6. Competence of Adult Newt RPE Cells for Differentiation into Nonretinal Cell Types

### 6.1. RPE Cell Conversion into Neuronal and Lentoid Phenotypes In Vitro

It is well known that in vitro culturing combined with targeted treatments makes it possible to reveal the silent potential of mammalian and human RPE cells for reprogramming into neural [64,65] or other (e.g., mesenchymal) directions [66]. The adult newt RPE is poorly culturable: it has a long lag phase in a medium with serum, retains for a long time its initial phenotype, and fails to enter the proliferative phase. Upon long-term incubation, however, cell depigmentation and formation of long cell processes may be revealed (personal observations). As reported in early studies, cells of the cultured newt RPE were not losing pigment granules but, after 40 days, transformed into eye lens cells arranged into lens-like structures (lentoids) [67]. During clonal culturing, some colonies after depigmentation also produced lentoids; other colonies reverted to the initial differentiation state, showing the ability to resume melanin synthesis; and only a small part of cells produced clones containing neuron-like cells with long processes that showed positive Bodian staining [68].

Isolated newt RPE cells in a serum-free medium did not proliferate but gradually lost their initial characteristics (pigmentation and RPE65 protein) and showed the signs of proneural differentiation, expressing pan-neural markers (neurofilament proteins and acetylated tubulin) and the Pax6 transcription factor [69]. In RPE organ cultures on filters, pigmented cells detached from the epithelial layer and gave rise to neuron-like progeny that expressed several neuron-specific proteins [70]. Notable effects were observed in our experiments with organotypic cultures of the RPE of *Pl. waltl* posterior eye segments in a 3D roller system: RPE cells dedifferentiated, began to express the NF-200 pan-neural marker, and produced an early retinal rudiment consisting of three rows of cells depigmented to different degrees, which closely resembled the NR rudiment formed in vivo [59]. None of the above experiments indicates that the newt RPE can differentiate into any extra ectodermal (in particular, mesenchymal) lineage, as is the case with cultures of the mammalian and human RPE [66]. They only confirm that its cells are highly competent for reprogramming into neural (retinal) cells. As for the expression of crystallins in the cultured RPE cells, it appears that their function in this case is related not to reprogramming and acquisition of new cell specialization but rather to the maintenance of cell homeostasis. It has been shown that crystallins play a role of chaperons for making and maintaining of topological and functional order of molecular complexes in a variety of cell types [71].

On the other hand, it is known that the human RPE has a limited neurogenic potential in vitro but is capable of epithelial–mesenchymal transition, expressing protein markers of the main mesenchymal cell types [66]. This phenomenon is responsible for certain eye pathologies, e.g., proliferative retinovitreopathy (PVR) [72]. An insight into the mechanisms of RPE plasticity is necessary for explaining the causes of PVR in higher vertebrates. As for prerequisites for reprogramming of the newt RPE, it is important to evaluate the possibilities and conditions of the conversion of its cells into mesenchymal lineages. Recently, it was shown for the first time that the change of RPE cell behavior and differentiation resembled very much those of PVR symptoms, also taking place in the newt eye [73]. When Pax6 was knocked down, RPE cells expressed key components of the epiretinal membrane characteristic of PVR. These results suggest Pax6 can be an important player that directs newt RPE cells to realize their retinogenic potential [73]. We also know that primary cultures of the newt RPE show upregulation of vimentin, an intermediate filament protein putatively characteristic of mesenchymal differentiation (personal observations).

### 6.2. Transformation of Newt RPE Cells into Melanophages In Vitro

A noteworthy feature common to RPE cells of newts and mammals is that they can change their morphological phenotype and convert into macrophage-like cells (melanophages) with a migratory behavior. This phenomenon in adult newts was described long ago at light and electron microscopic levels [35]. After the retina was removed or the optic nerve and blood vessels were cut, RPE cells acquired the ability to function as “cleaners” both within and outside the epithelial layer. The same has been repeatedly observed in subsequent experiments, particularly in 3D RPE tissue cultures [59]. Comparing this ability in the RPE of adult newts and albino rats cultured in vitro under similar conditions, we found that cells of the rat RPE also could detach from the layer and convert into melanophages, but the number of such cells was far smaller than in newts [8,59]. Melanophages perform the basic function of RPE cells, i.e., internalization and digestion of the shed outer segments of photoreceptors, and the phenomenon of cell conversion in this case cannot be interpreted as transdifferentiation. However, changes in cell phenotype and intercellular communications, migratory behavior, and the expression of phagocyte (macrophage)-specific markers imply modification of the initial gene expression pattern.

## 7. Specific Features of Cytoskeleton, Cell Contacts, and Extracellular Matrix Components in the Newt RPE

Characteristics of the cytoskeleton in the RPE reflect the epithelial type of this tissue, its functional specialization, and phenotypic plasticity [74]. We studied cytoskeletal components in the RPE of *Pl. waltl* in the norm and during reprogramming after retinectomy or retinal detachment [75,76,77]. For this purpose, we used antibodies against neurofilament protein NF-200 (a pan-neuronal marker) to reveal intermediate filaments and antibodies against cytokeratins as epithelial cell markers. The results showed that a high level of cytokeratin expression is characteristic of the normal RPE. It should be noted that we found published data that optic nerve astrocytes of *Pl. waltl* contain cytokeratins [78]. The authors interpret this as evidence that astrocytes retain their embryonic status. Apparently, the same may be true of newt RPE, the more so that its induction to reprogramming by isolation from the NR proved to result in rapid inhibition of cytokeratin expression [76,77]. The expression of these proteins was also inhibited immediately after isolation of *Pl. waltl* RPE and its dissociation into individual cells, i.e., under the effect of change in the conditions of cell microenvironment. During retinal regeneration, the expression of NF-200 was initiated already in the first cells that began to detach from the RPE layer and change their phenotype but still contained pigment granules in the cytoplasm [76]. These data indicate that the cytoskeleton and, in particular, intermediate filaments are active components of the program of change in the fate of RPE cells. Their reprogramming may be facilitated due to rapid switching to the transcription and translation of genes encoding pan-neuronal proteins and flexibility of this process including phosphorylation and dephosphorylation of intermediate filament proteins.

As in other vertebrates, cells of the newt RPE are connected by tight junctions located in the apical part of their lateral membrane [35,79]. It has also been shown that cells detaching from the RPE layer at the early stages of reprogramming retain gap junctional coupling with cells remaining in the layer [80]. Our experience in experiments with the newt RPE (which as a rule is readily dissociable into individual cells) and the data on de novo formation and changes in the localization of contacts between its cells upon their detachment from the layer suggest that these contacts are easily assembled and disassembled. This, in turn, is one more factor that facilitates reprogramming. However, this aspect of RPE biology, especially its molecular component, is as of yet poorly studied in Urodela, while relevant data on mammals are already available. For example, proteins of the signaling pathway associated with tight junctions (ZO and ZONAB) have been shown to play a regulatory role with respect to proliferation and differentiation of mouse RPE cells, with upregulation of ZONAB and downregulation of ZO-1 resulting in a change of the epithelial phenotype of these cells into a fibroblast-like phenotype [81]. It is also discussed how much the claudins, proteins of tight junctions, and such a type of cellular cooperation in the RPE differ from those of other epithelia. It is interesting to know also how they vary in different animal species [79]. Such differences in cell contact-associated proteins may also have an effect on success in RPE reprogramming in vivo.

A major role in the maintenance of cell differentiation is played by the microenvironment, particularly the behavior of extracellular matrix (ECM) molecules [82]. ECM operates not only as an adhesive substrate but also as a regulator of intracellular signals. The newt RPE has been characterized with respect to individual ECM components in the normal tissue and changes in their expression during its transdifferentiation. The vertebrate RPE is underlain by Bruch’s membrane, which is not a membrane proper but a complex lamina consisting of the RPE basement membrane, a dense layer of collagen and elastic fibers, and the basement membrane of the choroid [83]. Fibronectin is one of the main adhesion components of Bruch’s membrane [84]. In experiments with anti-fibronectin antibodies, specific immunofluorescence in the posterior segment of nonoperated newt (*Pl. waltl*) eyes was observed in the Bruch’s membrane, choroid, and sclera. On day 10 after retinal detachment, the fluorescence intensity decreased on the basal surface but increased on the lateral surfaces of adjoining RPE cells, which were entering the proliferation phase at that time [85]. A similar pattern of fibronectin distribution in the normal RPE and during its conversion was also observed by other authors [86]. Special attention should be given to the fact that the distribution and expression level of fibronectin proved to differ between the peripheral zone of the newt RPE, where its cells retain their tissue-specific phenotype, and its central zone, where cell conversion takes place [85]. This is evidence that conditions of the local microenvironment of RPE cells have a role in maintaining stability of their differentiation and that fibronectin is involved in this process. Its stabilizing effect is abolished soon after separation of RPE from NR, which may also be a factor of cell reprogramming. It should be noted that other components of ECM and basal membranes are also candidates for the role of regulators of RPE transdifferentiation. They include tenascin and N-CAM, whose expression changes in the course of newt RPE cell conversion [87] and laminin, which has proved to stimulate neural differentiation of frog RPE cells in vitro [88].

## 8. Transcription Factors and Signal Molecules Characteristic of Progenitor Cells in the Adult Newt RPE

The adult newt RPE in the course of in vivo reprogramming into NR cells passes through the stage of stem-like cell population and expresses genes coding for transcription factors known as regulators of early development and cell multipotency, such as *Pax6*, *Mitf*, *c-Myc*, *Klf4*, and *Sox2* [7]. In experiments on the eye tissues of adult *Pl. waltl* newts, we used RT-PCR and Western blotting to analyze the expression pattern of genes *Fgf2*, *Pax6*, *Six3*, *Otx2*, and *Pitx1*, which are involved in the regulatory network of eye field genes and play a key role in retinal development. The results show that the normal RPE (prior to cell reprogramming) contained transcripts and proteins encoded by homeobox genes of the *Pax*, *Otx2*, and *Pitx* families (*Pax6*, *Otx2*, and *Pitx1*/*Pitx2*), in addition to the *Rpe65* gene and its protein product considered above [12,13,37,89]. The expression of *Pax6* in the normal newt RPE deserves special attention in view of data on its role and place in the mechanisms of RPE cell reprogramming. One of the functions of this gene and its isoforms is related to self-renewal of multipotent eye cells. [90,91]. Analysis of neural progenitor cell population after *Pax6* gene silencing have revealed a decrease in the number of S-phase cells, increase of the number of cells which left cell cycle and, as a result, a disturbance of the process of cell differentiation. These events are related to changes in the expression of the target genes for PAX6 that stimulate passage through the cell cycle (*Ccnd1*, *Ccnd2*, *Ccnd3*) or inhibit it (*P27kip1*, *P27kip2*) and also of the SHH signal protein and transcription factors Vsx2, Nr2e1, and Plagl1 [92]. As shown previously, PAX6 expression blocking restricts the multipotency of retinal progenitor cells so that they differentiate into a single interneuronal lineage, producing an excess amount of amacrine cells [93]. We used different methods to study the expression of *Pax6* and its protein product during RPE cell reprogramming, including PCR, in situ hybridization, and immunohistochemical analysis [6,11,13]. A detailed analysis by highly sensitive PCR methods revealed the presence of *Pax6* mRNA and protein in normal RPE cells of adult *Pl. waltl* and *C. pyrrhogaster* newts [6,37]. In the former species, the level of *Pax6* transcripts in the RPE was lower than in the normal retina. It should be noted that, according to [94], *Pax6* has several transcripts (isoforms) produced as a result of alternative splicing, which are represented in the molecular profile of RPE in lower and higher vertebrates. The mechanism of *Pax6* splicing in vertebrates is evolutionary conserved and yields mainly two transcript variants, Pax6 and Pax6-5a, which can produce different protein isoforms with specific functions. For example, Pax6-5a has a role in inducing embryonic stem cells toward neural differentiation [95]. Each of the two isoforms can regulate its own set of target genes, but they also can have a cumulative effect on the expression of these genes [96,97]. Inami and co-authors (2016) have identified *Pах6* transcripts of different classes in *C. pyrrhogaster*. Splice variants of classes 1 and 2 (v1 and v2) are activated at early stages of NR regeneration but remain silent in the normal RPE, with class 1 variants being expressed in the normal NR [97]. By means of inhibitor analysis, these authors have also shown that *Pax6* expression is controlled via signaling pathways that differ from the canonical MEK-ERK pathway responsible for the initiation of RPE cell proliferation. Of interest is also the expression pattern of the Sox2 protein at the initial stages of RPE reprogramming. Unexpectedly, it has proved to be similar to that of the Pax6 protein: Sox2 immunoreactivity appears in a few still pigmented but already transdifferentiating RPE cells and markedly increases in the regenerating retinal rudiment [7].

If our premise is that the RPE and other newt eye tissues retain molecular attributes of developmental regulation, we should take into account the possible role of homeobox gene *Chx10* in RPE reprogramming. The transcription factor encoded by this gene can suppress melanin accumulation in the presumptive chick RPE and promote NR differentiation; i.e., it can regulate processes that involve the expression of genes relevant to retinal development: *Sox2*, *Six3*, *Rx1*, and *Optx2*. In accordance with the results, *Chx10* gene expression could be responsible for determination of cell identity in the complex structure of developing retinal anlage, acting upstream in the cascade of transcription [98]. There are data that *Chx10* is expressed in RPE-derived neuroblasts of the regenerating retinal rudiment [6,20] and it appears expedient to analyze whether reprogramming-associated transient misexpression of this gene takes place in the normal RPE of adult newts.

It is currently accepted that retinal progenitor cells are competent to develop in two different (but closely related) ways, to become RPE or neural retina cells, and this choice is under regulation by the link between fibroblast growth factor (FGF) and microphthalmia-associated transcription factor (MITF) [99,100]. The FGF signaling pathway is known to play a key role in the neural retina formation during development and regeneration [53,101,102]. Basic fibroblast growth factor Fgf2 has five isoforms with different functions and subcellular localization. The 18 kDa isoform is mainly cytosolic and operates through cell surface receptors, while isoforms with higher molecular weights (22, 22.5, 24, and 34 kDa) are predominantly located in the nucleus and function independently of the transmembrane receptor pathway [103]. However, the endogenous 18 kDa isoform has been revealed in the nucleoplasm and nucleolus [104] and shown to directly regulate rRNA transcription, interacting with nucleolar transcription factors [105]. Thus, Fgf2 signaling can regulate rRNA transcription via both intra- and extracellular pathways regulation [106]. It is noteworthy in this context that the expression of genes encoding the basic components of the FGF2 signaling pathway—Fgf2 itself and its receptor Fgfr2—as well as their products have been detected in the normal newt RPE [22]. The role of this pathway in RPE reprogramming remains unclear, but it has been found that intensification of its function correlates mainly with progression of cell proliferation rather than with initiation of reprogramming [22]. The Notch signaling pathway is also important for the initiation and early progression of newt RPE cell reprogramming [20,21]. It has been shown that Notch and its ligands are upregulated immediately after damage, which is usually regarded as a reliable indicator of regeneration in sensory systems [107]. The involvement of Notch-1 signaling at successive stages of RPE reprogramming in adult *C. pyrrhogaster* newts has been studied in detail using Notch-1 cDNA probes derived from neural plate to tailbud stage embryos [20]. It has been found that Notch-1 receptors are already expressed at the stage of cell displacement from the RPE layer, with their expression increasing in the course of cell conversion and growth of the amplifying cell population of the regenerating retinal rudiment. Results obtained suggest that Notch-1 signaling pathway regulating neurogenesis during development is likely recruited for similar functions in retinal regeneration. Moreover, as shown by PCR analysis, RPE cells of the adult newt express some genes involved in Notch signaling (such as *Hes-1*, *neurogenin1*, and occasionally *Delta-1*) on postoperative day 0 and that these genes are apparently upregulated before the onset of Notch-1 expression [20]. That, in turn, means that Notch-1 pathway is partially activated prior to the beginning of the way to RPE cell type conversion. It is important in this context that the same authors performed RT-PCR analysis of single freshly isolated RPE cells, and the results proved to be consistent with those of PCR with eye cups of postoperative day 0, showing that RPE cells expressed both Hes-1 and Ngn-1. Summarizing these results, it may be hypothesized that the components of the proneural Notch signaling pathways are initially expressed in normal RPE cells of adult newts or that separation of RPE cells from each other is sufficient for triggering this pathway. Anyway, the above data show that the expression of Notch-1 pathway components in the adult newt RPE may be included in the set of factors accounting for its high competence for reprogramming into retinal cells.

On the other hand, analysis of the adult rat RPE for the expression of transcription factors, signaling pathway components, and cytoskeletal proteins characteristic of retinal stem/progenitor cells (Pax6, NeuroD, Notch, Musashi, βIII-tubulin, Dcx, Nestin) showed that none of them was expressed in the freshly isolated RPE, except for transcription factor Hes1. However, all these markers were reliably detected in the RPE cultured in vitro, after the third passage under permissive conditions for differentiation in neuronal direction [108]. It may well be that new, more sensitive research methods will reveal the expression of certain markers of early eye development and proneuronal differentiation in the RPE of adult mammals and that differences in their expression between newts and mammals will prove to be mainly quantitative rather than qualitative. It is also probable that the pattern of genetic and epigenetic regulation of their expression differs between these groups.

## 9. Epigenetic Factors in Cell of Normal RPE and at Early Stages of Reprogramming

### 9.1. Chromatin Reorganization in Cells of Adult Newt RPE at the Onset of Reprogramming

Changes in the RPE occur immediately after its detachment from the NR, intraocular pressure relief, and relaxation of tension within the layer [8]. Some cells leave the RPE layer and change morphologically: they acquire an irregular oval shape, the relative volume of the nucleus increases, and chromatin rearrangements take place. The chromatin state—the packaging of DNA with histone and nonhistone proteins—has a profound effect on gene expression and can contribute to the establishment and maintenance of cell identities. It is becoming evident that the dynamic composition of chromatin plays an important role in the regulation of activities of enzymes [109]. Genome reprogramming in RPE cells is accompanied by chromatin rearrangements and changes in 3D organization of chromosomal loci, which, in turn, play a major role in the regulation of gene activity. The pattern of these rearrangements in newt RPE cells during reprogramming and their role in initiating a transcriptional program for retinogenic differentiation in these cells have not been studied systematically. We made an attempt to characterize the state of chromatin in RPE cells in preliminary experiments on the model of RPE transdifferentiation in *Pl. waltl* newts after different kinds of damage disrupting the RPE–NR contact (bright light irradiation, mechanical retinal detachment, or retinectomy). The material was stained with toluidine blue and DAPI (4′,6-diamidino-2-phenylindole) and analyzed in semithin sections at high magnification [23,110]. In cells of the normal RPE, the nuclei contained mainly diffuse chromatin composed of granular elements and fibers. Small regions of condensed chromatin (10–20 per nucleus) were distributed largely at the nuclear periphery and often attached to the nuclear membrane (parietal heterochromatin). Initiation of RPE cell reprogramming after the loss of contact with photoreceptors was accompanied by chromatin displacement to the nuclear center and a significant increase in the size of condensed chromatin regions, with consequent change in the ratio of condensed to diffuse chromatin in favor of the former. Such regions were structurally segregated into discrete large blocks (chromocenters) (Figure 1B). Thus, RPE cells at the very onset of reprogramming, long before entering the S-phase, already show chromatin condensation, which is indicative of transcriptional repression rather than activation. The observed changes in the degree of chromatin condensation and distribution pattern occur before the cells lose specific products of their initial differentiation (such as melanin), whose synthesis is already blocked [10,33]. RPE cells increasingly lose the traits of initial differentiation as they start to proliferate. Both these processes occur in parallel, against the background of molecular genetic events considered above [6,7,11,37]. It should be emphasized that a distinctive feature of the early stage of reprogramming is that the expression of genes characteristic of the initial RPE cells is combined with upregulation of genes responsible for their new, proneuronal differentiation. Apparently, the described changes in chromatin and nuclear membrane are related to these processes and transition to the stage of active proliferation.

In recent years, significant progress has been made in understanding the roles of histone modifications and chromatin remodeling in cell differentiation [109]. Such data on the newt RPE are absent, but it is already known that the embryonic linker histone B4 is expressed and required during transdifferentiation of iris pigment epithelial cells into lens cells during lens regeneration in newts [111]. As found in our early experiments, newt RPE cells at the onset of reprogramming, before entering active proliferation, show a significant increase in total protein production (including nonhistone proteins); according to quantitative estimates based on ^3^H-tryptophan incorporation, the rate of protein synthesis increases twofold at this stage [60].

### 9.2. Nucleostemin Expression

Nucleostemin (Ns), or guanine nucleotide binding protein-like 3 (GNL3/Gnl3), is one of nucleolar proteins that plays an important role in epigenetic control of cellular processes. We have studied its expression in order to characterize in more detail the epigenetic state of the RPE. Nucleostemin (a marker of low-differentiated cells) has been identified in pluripotent embryonic stem cells and low-differentiated neural cells in both vertebrates and invertebrates [112,113,114,115]. It has various functions and is involved in the regulation of RNA polymerase I activity, transcription, chromatin structure, etc. [116,117,118,119,120,121]. We found nucleostemin in cells of normal eye tissues, including the RPE of adult *Pl. waltl* newts [24], where the expression of its gene (*Ns*) was revealed by PCR. The sequencing of amplicons confirmed their homology to the target gene sequence. The expression of this gene and other markers of low-differentiated cells in RPE cells may be indicative of its involvement in the molecular mechanisms accounting for the plasticity and retinogenic potential of this tissue. The expression of the nucleostemin gene was also revealed in differentiated pigment epithelial cells of the iris in *C. pyrrhogaster* newts. These cells were capable of conversion into lens cells [122]. Attention should also be given to the Musashi1 protein (Msi1) expressed in the nuclei of normal RPE cells. Upon retinectomy, Msi1 is localized in the cytoplasm and nucleus of RPE-derived cells and maintains its expression during all phases of reprogramming, including differentiation of retinal cell types. There is a suggestion that Msi1 plays important but still unknown role(s) in the process of RPE cell phenotype conversion and in posttranslational regulation, in particular [123].

On the whole, it may be concluded that further studies are needed to gain an insight into the epigenetic aspect of in vivo reprogramming as related to its specific features in specialized somatic cells such as the newt RPE cells.

## 10. Conclusions

An important mechanism underlying the regeneration capacity of vertebrates is natural cell reprogramming, i.e., the conversion of differentiated cells into a different cell type. The known examples of this process are few, and their analysis (along with the search for new relevant experimental models) is necessary for developing approaches to stimulate regeneration in vivo based on adult reserve cells. To this end, in turn, it is important to gain an insight into not only the conditions leading to destabilization of cell differentiation but also into the competence of cells to respond to appropriate stimuli. The conversion of retinal pigment epithelium (RPE) cells into neural retinal cells in adult newts (Urodela) is a classic example of natural cell reprogramming in vivo. This process leads to perfect regeneration of the retina even after its surgical removal. In view of the results of our long-term studies, we have recently focused on the search for specific cellular and molecular features of the newt RPE that can be related to its unique ability to transform into neurons and glial cells and give rise to de novo formation of the retina. As a result, several lines of evidence have been obtained that, in the aggregate, indicate that RPE cells may combine the properties of functionally specialized cells and their low-differentiated progenitors. Thus, RPE cells in situ express not only the initial cell type-specific markers (melanin, RPE65 protein, transcription factors Otx2, Mitf, etc.) but also the transcription factors, signal molecules, and marker proteins of neuronal progenitor cells (FGF2, Pax6, Ns, E-NTPDase, с-myc, etc). Moreover, the cells of adult newt RPE show persistent, low-level (restrained) proliferative activity, can quickly enter the amplification phase, and are capable of cytoskeleton remodeling, with epithelial cytokeratins being substituted by neurofilament proteins under the effect of changes in the extracellular matrix. Epigenetic processes, particularly chromatin remodeling, also contribute to the competence of RPE cells for reprogramming. Some of the factors determining such competence have been described for other animals in which no adult RPE reprogramming is observed. However, it may well be that all the relevant properties as a whole are essential only to representatives of the order Urodela, which are known to be pedomorphic (i.e., to retain juvenile features in the adult age). We have revealed certain properties of newt RPE cells that facilitate their reprogramming, and this information can contribute to the development of approaches to experimental “rejuvenation” of cells, i.e., to the reversal of their terminal differentiation providing for the involvement of these cells in the processes of regeneration and repair.

## References

[1] Sparrrow J.R., Hicks D., Hamel C.P. (2010). The Retinal Pigment Epithelium in Health and Disease. Curr. Mol. Med..

[2] Fuhrmann S., Zou C., Levine E.M. (2014). Retinal pigment epithelium development, plasticity, and tissue homeostasis. Exp. Eye Res..

[3] Hasegawa M. (1958). Restitution of the eye after removal of the retina and lens in the newt *Triturus pyrrhogaster*. Embryologia.

[4] Stroeva O.G., Mitashov V.I. (1983). Retinal pigment epithelium: proliferation and differentiation during development and regeneration. Int. Rev. Cytol..

[5] Mitashov B.I. (1996). Mechanisms of retina regeneration in urodele. Int. J. Dev. Biol..

[6] Chiba C., Mitashov V.I., Chiba C. (2007). Cellular and molecular events in the adult newt retinal regeneration. Strategies for Retinal Tissue Repair and Regeneration in Vertebrates: From Fish to Human.

[7] Islam M.R., Nakamura K., Casco-Robles M.M., Kunahong A., Inami W., Toyama F., Maruo F., Chiba C. (2014). The newt reprograms mature RPE cells into a unique multipotent state for retinal regeneration. Sci. Rep..

[8] Grigoryan E.N., Davies J. (2012). Shared triggering mechanisms of retinal regeneration in lower vertebrates and retinal rescue in higher ones. Tissue Regeneration—From Basic Biology to Clinical Application.

[9] Okada T.S. (1991). Transdifferentiation: Flexibility in Cell Differentiation.

[10] Grigoryan E.N. (1998). Cytological bases of transdifferentiation in eye tissues of vertebrates. Extended Abstract of Doctoral (Biol.) Dissertation.

[11] Markitantova Y.V., Makar’ev E.O., Smirnova Y.A., Zinov’eva R.D., Mitashov V.I. (2004). Analysis of the expression pattern of regulatory genes Pax6, Prox1, and Six3 during regeneration of eye structure in newt. Biol. Bull. (Moscow).

[12] Markitantova Y.V., Avdonin P.P., Grigoryan E.N., Zinov’eva R.D. (2010). Identification of the Pitx1 embryogenesis regulatory gene in a regenerating newt retina. Dokl. Biol. Sci..

[13] Avdonin P.P., Grigoryan E.N., Markitantova Y.V. (2010). Transcriptional factor Pitx2: Localization during triton retina regeneration. Biol. Bull. (Moscow).

[14] Nakamura K., Rafiqul M.R., Takayanagi M., Yasumuro H., Inami W., Kunahong A., Casco-Robles R.M., Toyama F., Chiba C. (2014). A Transcriptome for the Study of Early Processes of Retinal Regeneration in the Adult Newt, *Cynops pyrrhogaster*. PLoS One.

[15] Lopashov G.V., Stroeva O.G. (1961). Morphogenesis of the vertebrate eye. Advance in Morphogenesis.

[16] Heavner W., Pevny L. (2012). Eye Development and Retinogenesis. Cold Spring Harb. Perspect. Biol..

[17] Mitashov V.I. (2007). Expression of regulatory and tissue-specific genes controlling regenerative potencies of eye tissues in vertebrates. Russ. J. Dev. Biol..

[18] Chiba C., Hoshino A., Nakamura K., Susaki K., Yamano Y., Kaneko Y., Kuwata O., Maruo F., Saito T. (2006). Visual cycle protein RPE65 persists in new retinal cells during retinal regeneration of adult newt. J. Comp. Neurol..

[19] Sakami S., Hisatomi O., Sakakibara S., Liu J., Reh T.A., Tokunaga F. (2005). Downregulation of Otx2 in the dedifferentiated RPE cells of regenerating newt retina. Brain Res. Dev. Brain Res..

[20] Nakamura K., Chiba C. (2007). Evidence for Notch signaling involvement in retinal regeneration of adult newt. Brain Res..

[21] Kaneko Y., Hirota K., Matsumoto G., Hanyu Y. (2001). Expression pattern of a newt Notch homologue in regenerating newt retina. Brain Res. Dev. Brain Res..

[22] Markitantova Y.V., Avdonin P.P., Grigoryan E.N. (2014). FGF2 signaling pathway components in tissues of the posterior eye sector in the adult newt *Pleurodeles waltl*. Biol. Bull. (Moscow).

[23] Markitantova Y.V., Avdonin P.P., Grigoryan E.N. (2014). Nucleostemin expression during in situ reprogramming of eye pigment epithelium cells during retina regeneration in an adult newt. Cell Tissue Biol..

[24] Markitantova Y.V., Avdonin P.P., Grigoryan E.N. (2015). Identification of the Gene Encoding Nucleostemin in the Eye Tissues of *Pleurodeles waltl*. Biol. Bull. (Moscow).

[25] Fuhrmann S. (2010). Eye Morphogenesis and Patterning of the Optic Vesicle. Curr. Top. Dev. Biol..

[26] Adler R., Canto-Soler M.V. (2007). Molecular mechanisms of optic vesicle development: Complexities, ambiguities and controversies. Dev. Biol..

[27] Raviv S., Bharti K., Rencus-Lazar S., Cohen-Tayar Y., Schyr R., Evantal N., Meshorer E., Zilberberg A., Idelson M., Reubinoff B. (2014). PAX6 Regulates Melanogenesis in the Retinal Pigmented Epithelium through Feed-Forward Regulatory Interactions with MITF. PLOS Genet..

[28] Grigoryan E.N. (2016). High regenerative ability of tailed amphibians (Urodela) as a result of the expression of juvenile traits by mature animals. Russ. J. Dev. Biol..

[29] Grigoryan E.N., Ivanova N.I., Poplinskaya V.A. (1996). The discovery of a new internal sources of neural retinal regeneration after its detachment in newts. Morphological and quantitative research. Izv. Akad. Nauk. Ser. Biol..

[30] Grigoryan E.N., Chiba C. (2007). Alternative intrinsic cell sources for neural retina regeneration in adult urodelean amphibians. Strategies for Retinal Tissue Repair and Regeneration in Vertebrates: From Fish to Human.

[31] Mitashov V.I. (1997). Retinal regeneration in amphibians. Int. J. Dev. Biol..

[32] Goto T., Tokunaga F., Hisatomi O. (2012). Hematological- and Neurological-Expressed Sequence 1 Gene Products in Progenitor Cells during Newt Retinal Development. Stem Cells Int..

[33] Grigorian E.N., Mitashov V.I. (1979). Radioautographic investigation of proliferation and melanin synthesis in retinal pigment epithelium cells during eye regeneration in the newt. Ontogenez.

[34] Schraermeyer U., Heimann K. (1999). Current understanding of the role of retinal pigment epithelium and its pigmentation. Pigment Cell Res..

[35] Keefe J.R. (1973). An analysis of urodelian retinal regeneration. J. Exp. Zool..

[36] Moiseyev G., Chen Y., Takahashi Y., Wu B.X., Ma J.-X. (2005). RPE65 is the isomerohydrolase in the retinoid visual cycle. Proc. Natl. Acad. Sci. USA.

[37] Avdonin P.P., Markitantova Y.V., Zinov’eva R.D., Mitashov V.I. (2008). Expression of regulatory genes Pax6, Otx2, Six3, and FGF2 during newt retina regeneration. Biol. Bull. (Moscow).

[38] Rattner A., Toulabi L., Williams J., Yu H., Nathans J. (2008). The genomic response of the retinal pigment epithelium to light damage and retinal detachment. J. Neurosci..

[39] Fujimura N., Taketo M.M., Mori M., Korinek V., Kozmik Z. (2009). Spatial and temporal regulation of WNT/beta-catenin signaling is essential for development of the retinal pigment epithelium. Dev. Biol..

[40] Bharti K., Gasper M., Ou J., Brucato M., Clore-Gronenborn K., Pickel J., Arnheiter H. (2012). A Regulatory Loop Involving PAX6, MITF, and WNT Signaling Controls Retinal Pigment Epithelium Development. PLoS Genet..

[41] Blenkinsop T.A., Salero E., Stern J.H., Temple S. (2013). The culture and maintenance of functional retinal pigment epithelial monolayers from adult human eye. Method. Mol. Biol..

[42] Steinfeld J., Steinfeld I., Coronato N., Hampel M.-L., Layer P.G., Araki M., Vogel-Höpker A. (2013). RPE specification in the chick is mediated by surface ectoderm derived BMP and Wnt signaling. Development.

[43] Westenskow P.D., McKean J.B., Kubo F., Nakagawa S., Fuhrmann S. (2010). Ectopic Mitf in the Embryonic Chick Retina by Co-transfection of b-Catenin and Otx2. Invest. Ophthalmol. Vis. Sci..

[44] Burke J.M. (2008). Epithelial phenotype and the RPE: is the answer blowing in the WNT?. Prog. Ret. Eye Res..

[45] Aulicino F., Theka I., Ombrato L., Lluis F., Cosma P.M. (2014). Temporal perturbation of the Wnt signaling pathway in the control of cell reprogramming is modulated by TGFI. Stem Cell Rep..

[46] Fang Y., Cho K-S., Tchedre K., Lee S.W., Guo C., Kinouchi H., Fried S., Sun X., Chen D.F. (2013). Ephrin-A3 Suppresses Wnt Signaling to Control Retinal Stem Cell Potency. Stem Cells.

[47] Ahmad I., Tang L., Pham H. (2000). Identification of neural progenitors in the adult mammalian eye. Biochem. Biophys. Res. Commun..

[48] Tropepe V., Coles B.L., Chiasson B.J., Horsford D.J., Elia A.J., McInnes R.R., van der Kooy D. (2000). Retinal stem cells in the adult mammalian eye. Science.

[49] Nishihara D., Yajima I., Tabata H., Nakai M., Tsukiji N., Katahira T., Takeda K., Shibahara S., Nakamura H., Yamamoto H. (2012). Otx2 is involved in the regional specification of the developing retinal pigment epithelium by preventing the expression of Sox2 and Fgf8, factors that induce neural retina differentiation. PLoS ONE.

[50] Martinez-Morales J.R., Dolez V., Rodrigo I., Zaccarini R., Leconte L., Bovolenta P., Saule S. (2003). Otx2 activates the molecular network underlying retinal pigment epithelium differentiation. J. Biol. Chem..

[51] Li P., Sun X., Ma Z., Liu Y., Jin Y., Ge R., Hao L., Ma Y., Han S., Sun H. (2016). Transcriptional Reactivation of *OTX2, RX1* and *SIX3* during Reprogramming Contributes to the Generation of RPE Cells from Human iPSCs. Int. J. Biol. Sci..

[52] Zhang X.M., Yang X.J. (2001). Temporal and spatial effects of sonic hedgehog signaling in chick eye morphogenesis. Dev. Biol..

[53] Muller F., Rohrer H., Vogel-Hopker A. (2007). Bone morphogenetic proteins specify the retinal pigment epithelium in the chick embryo. Development.

[54] Spence J.R., Aycinena J.C., Del Rio-Tsonis K. (2007). Fibroblast growth factor-hedgehog interdependence during retina regeneration. Dev. Dyn..

[55] Sakami S., Etter P., Reh T.A. (2008). Activin signaling limits the competence for retinal regeneration from the pigmented epithelium. Mech. Dev..

[56] Novikova Y.P., Poplinskaya V.A., Aleinikova K.S., Grigoryan E.N. (2008). A study of the localization and accumulation of S-phase cells in the retina of newt *Pleurodeles waltl* after experimental pigment epithelial detachment. Russ. J. Dev. Biol..

[57] Fischer A.J., Reh T.A. (2000). Identification of a proliferating marginal zone of retinal progenitors in postnatal chickens. Dev. Biol..

[58] Al-Hussaini H., Kam J.H., Vugler A., Semo M., Jeffery G. (2008). Mature retinal pigment epithelium cells are retained in the cell cycle and proliferate in vivo. Mol. Vis..

[59] Novikova Y.P., Aleinikova K.S., Poplinskaya V.A., Grigoryan E.N. (2010). The retinal pigment epithelial cells of the adult newt and rat under conditions of in vitro organotypic culture. Biol. Bull. (Moscow).

[60] Mitashov V.I. (1980). Patterns of changes in mitotic cycles during cell transformation and regeneration in lower vertebrates. Tsitologiia.

[61] Yun M.H., Gates P.B., Brockes J.P. (2014). Sustained ERK Activation Underlies Reprogramming in Regeneration Competent Salamander Cells and Distinguishes Them from Their Mammalian Counterparts. Stem Cell Rep..

[62] Mizuno A., Yasumuro H., Yoshikawa T., Inami W., Chiba C. (2012). MEK-ERK signaling in adult newt retinal pigment epithelium cells is strengthened immediately after surgical induction of retinal regeneration. Neurosci. Lett..

[63] Yoshikawa T., Mizuno A., Yasumuro H., Inami W., Vergara M.N., Del Rio-Tsonis K., Chiba C. (2012). MEK–ERK and heparin susceptible signaling pathways are involved in cell cycle entry of the wound edge retinal pigment epithelium cells in the adult newt. Pigment Cell Melanoma Res..

[64] Kuznetsova A.V., Grigoryan E.N., Aleksandrova M.A. (2011). Adult human retinal pigment epithelial cells—A potential source of cells for regeneration retina. Tsitologiia.

[65] Milyushina L.A., Verdiev B.I., Kuznetsova A.V., Aleksandrova M.A. (2012). Expression of pluripotent and retinal markers in pigment retinal epithelium in adult human eye in vitro. Klet. Tekhnol. Biol. Med..

[66] Salero E., Blenkinsop T.A., Corneo B., Harris A., Rabin D., Stern J.H., Temple S. (2012). Adult human RPE can be activated into a multipotent stem cell that produces mesenchymal derivatives. Cell Stem Cell.

[67] Eguchi G., Ebert J.D., Okada T.S. (1979). “Transdifferentiation” in pigmented epithelial cells of vertebrate eyes in vitro. Mechanisms of Cell Change.

[68] Eguchi G. (1993). Lens transdifferentiation in the vertebrate retinal pigmented epithelial cell. Prog. Retin. Eye Res..

[69] Susaki K., Chiba C. (2007). MEK mediates in vitro neural transdifferentiation of the adult newt retinal pigment epithelium cells: Is FGF2 an induction factor?. Pigment Cell Res..

[70] Ikegami Y., Mitsuda S., Araki M. (2002). Neural cell differentiation from retinal pigment epithelial cells of the newt: An organ culture model for the urodele retinal regeneration. J. Neurobiol..

[71] Slingsby C., Wistow G.J. (2014). Functions of crystallins in and out of lens: Roles in elongated and post-mitotic cells. Prog. Biophys. Mol. Biol..

[72] Heidenkummer H.P., Kampik A. (1991). Comparative immunohistochemical studies of epiretinal membranes in proliferative vitreoretinal diseases. Fortschr. Ophthalmol..

[73] Casco-Robles M.M., Islam M.R., Inami W., Tanaka H.V., Kunahong A., Yasumuro H., Hanzawa S., Casco-Robles R.M., Toyama F., Maruo F. (2016). Turning the fate of reprogramming cells from retinal disorder to regeneration by Pax6 in newts. Sci. Rep..

[74] Bonilha V.L. (2013). Retinal pigment epithelium (RPE) cytoskeleton in vivo and in vitro. Exp. Eye Res..

[75] Grigoryan E.N., Anton H.J. (1993). The appearance and distribution of the NF-200 neurofilament protein in transdifferentiating cells of the pigment epithelium and in other eye cells during retinal regeneration in newts. Ontogenez.

[76] Grigoryan E.N., Anton H.J. (1995). An analysis of keratin expression in the cells of the retinal pigment epithelium during transdifferentiation in newts. Ontogenez.

[77] Grigoryan E.N. (1995). Complete retinal detachment causes changes in the expression of cytokeratins in retinal pigment epithelium cells in newts. Izv. Akad. Nauk. Ser. Biol..

[78] Rungger-Brandle E., AchtstZitter T., Franke W.W. (1989). An epithelium-type cytoskeleton in a glial cell astrocytes of an amphibian optic nerve contain cytokeratin filaments and are connected by desmosomes. J. Cell Biol..

[79] Rizzolo L.J., Peng S., Luo Y., Xiao W. (2011). Integration of tight junctions and claudins with the battier functions of the retinal pigment epithelium. Prog. Retin. Eye Res..

[80] Chiba C., Saito T. (2000). Gap junctional coupling between progenitor cells of regenerating retina in the adult newt. J. Neurobiol..

[81] Georgiadis A., Tschernutter M., Bainbridge J.W., Balaggan K.S., Mowat F., West E.L., Munro P.M., Thrasher A.J., Matter K., Balda M.S. (2010). The tight junction associated signaling proteins ZO-1 and ZONAB regulate retinal pigment epithelium homeostasis in mice. PLoS ONE.

[82] Hausman R.E. (2007). Ocular extracellular matrices in development. Prog. Retin. Eye Res..

[83] Hiscott P., Sheridan C., Magee R.M., Grierson I. (1999). Matrix and the retinal pigment epithelium in proliferative retinal disease. Prog. Retin. Eye Res..

[84] Mohan P.S., Spiro R.G. (1986). Macromolecular organization of basement membranes. J. Biol. Chem..

[85] Grigoryan E.N., Dol’nikova A.E., Belkin V.M. (1990). Fibronectin distribution during the transdifferentiation and proliferation of eye cells after retinal detachment and removal of the crystalline lens in newts. Ontogenez.

[86] Ortiz J.R., Vigny M., Courtois Y., Jeanny J.-C. (1992). Immunocytochemical study of extracellular matrix components during lens and neural retina regeneration in the adult newt. Exp. Eye Res..

[87] Mitashov V.I., Arsanto J.P., Markitantova Y.V., Thouveny Y. (1995). Remodeling processes during neural retinal regeneration in adult urodeles: An immunohistochemical survey. Int. J. Dev. Biol..

[88] Reh T.A., Nagy T., Gretton H. (1987). Retinal pigmented epithelial cells induced to transdifferentiate to neurons by laminin. Nature.

[89] Grigoryan E.N., Markitantova Y.V., Avdonin P.P., Radugina E.A. (2013). Study of regeneration in amphibians in age of molecular-genetic approaches and methods. Russ. J. Genet..

[90] Oron-Karni V., Farhy C., Elgart M., Marquardt T., Remizova L., Yaron O., Xie Q., Cvekl A., Ashery-Padan R. (2008). Dual requirement for Pax6 in retinal progenitor cells. Development.

[91] Debbio C.B.D., Peng X., Xiong H., Ahmad I. (2013). Adult ciliary epithelial stem cells generate functional neurons and differentiate into both early and late born retinal neurons under non-cell autonomous influences. BMC Neurosci..

[92] Farhy C., Elgart M., Shapira Z., Oron-Karni V., Yaron O., Menuchin Y., Rechavi G., Ashery-Padan R. (2013). *Pax6* Is Required for Normal Cell-Cycle Exit and the Differentiation Kinetics of Retinal Progenitor Cells. PLoS ONE.

[93] Marquardt T., Ashery-Padan R., Andrejewski N., Scardigli R., Guillemot F., Gruss P. (2001). Pax6 is required for the multipotent state of retinal progenitor cells. Cell.

[94] Lakowski J., Majumder A., Lauderdale J.D. (2007). Mechanisms controlling Pax6 isoform expression in the retina have been conserved between teleosts and mammals. Dev. Biol..

[95] Shimizu N., Watanabe H., Kubota J., Wu J., Saito R., Yokoi T., Era T., Iwatsubo T., Watanabe T., Nishina S. (2009). Pax6-5a promotes neuronal differentiation of murine embryonic stem cells. Biol. Pharm. Bull..

[96] Bandah D., Swissa T., Ben-Shlomo G., Banin E., Ofri R., Sharon D. (2007). A Complex Expression Pattern of Pax6 in the Pigeon Retina. Invest. Ophthalmol. Vis. Sci..

[97] Inami W., Islam M.R., Nakamura K., Yoshikawa T., Yasumuro H., Casco-Robles M.M., Toyama F., Maruo F., Chiba C. (2016). Expression of Two Classes of *Pax6* Transcripts in Reprogramming Retinal Pigment Epithelium Cells of the Adult Newt. Zool. Sci..

[98] Wang Z., Yasugi S., Ishii Y. (2016). ChxC10 functions as a regulator of molecular pathways controlling the regional identity in the primordial retina. Dev. Biol..

[99] Hyer J., Mima T., Mikawa T. (1998). FGF-1 patterns the optic vesicle by directing the placement of the neural retina domain. Development.

[100] Nguyen M., Arnheiter H. (2000). Signaling and transcriptional regulation in early mammalian eye development: A link between FGF and MITF. Development.

[101] Park C.M., Hollenberg M.J. (1989). Basic fibroblast growth factor induces retinal regeneration in vivo. Dev. Biol..

[102] Martinez-Morales J.R., Del Bene F., Nica G., Hammerschmidt M., Bovolenta P., Wittbrodt J. (2005). Differentiation of the vertebrate retina is coordinated by an FGF signaling center. Dev. Cell.

[103] Delrieu I. (2000). The high molecular weight isoforms of basic fibroblast growth factor (FGF-2): An insight into an intracrine mechanism. FEBS Lett..

[104] Claus P., Doring F., Gringel S., Muller-Ostermeyer F., Fuhlrott J., Kraft T., Grothe C. (2003). Differential intranuclear localization of fibroblast growth factor-2 isoforms and specific interaction with the survival of motoneuron protein. J. Biol. Chem..

[105] Sheng Z., Liang Y., Lin C.-Y., Comai L., Chirico W.J. (2005). Direct Regulation of rRNA Transcription by Fibroblast Growth Factor 2. Mol. Cell. Biol..

[106] Bouche G., Gas N., Prats H., Baldin V., Tauber J.P., Teissie J., Amalric F. (1987). Basic fibroblast growth factor enters the nucleolus and stimulates the transcription of ribosomal genes in ABAE cells undergoing G_0_→G_1_ transition. Proc. Natl. Acad. Sci. USA.

[107] Bermingham-McDonogh O., Reh T.A. (2011). Regulated Reprogramming in the Regeneration of Sensory Receptor Cells. Neuron.

[108] Engelhardt M., Bogdahn U., Aigner L. (2005). Adult retinal pigment epithelium cells express neural progenitor properties and the neuronal precursor protein doublecortin. Brain Res..

[109] Chen T., Dent S.Y.R. (2014). Chromatin modifiers and remodellers: Regulators of cellular differentiation. Nat. Rev. Genet..

[110] Markitantova Y.V., Poplinskaya V.A., Grigoryan E.N. Chromatin reorganization during early stages of the RPE reprogramming in regenerating *Pleurodeles waltl* newt retina. Proceedings of the International Conference Chromosome 2015.

[111] Maki N., Suetsugu-Maki R., Sano S., Nakamura K., Nishimura O., Tarui H., Del Rio-Tsonis K., Ohsumi K., Agata K., Tsonis P.A. (2010). Oocyte-type linker histone B4 is required for transdifferentiation of somatic cells in vivo. FASEB J..

[112] Tsai R.Y.L., McKay R.D. (2005). A multistep, GTP-driven mechanism controlling the dynamic cycling of nucleostemin. J. Cell Biol..

[113] Ma H., Pederson T. (2008). Nucleostemin: A multiplex regulator of cell-cycle progression. Trends Cell Biol..

[114] Rosby R., Cui Z., Rogers E., deLivron M.A., Robinson V.L., DiMario P.J. (2009). Knockdown of the *Drosophila* GTPase nucleostemin 1 impairs large ribosomal subunit biogenesis, cell growth, and midgut precursor cell maintenance. Mol. Biol. Cell.

[115] Paridaen J.T., Janson E., Utami K.H., Pereboom T.C., Essers P.B., van Rooijen C., Zivkovic D., Macinnes A.W. (2011). The nucleolar GTP-binding proteins Gnl2 and nucleostemin are required for retinal neurogenesis in developing zebrafish. Dev. Biol..

[116] Lin T., Ibrahim W., Peng C.-Y., Finegold M.J., Tsai R.Y.L. (2013). A novel role of nucleostemin in maintaining the genome integrity of dividing hepatocytes during mouse liver development and regeneration. Hepatology.

[117] Tsai R.Y.L., McKay R.D. (2002). A nucleolar mechanism controlling cell proliferation in stem cells and cancer cells. Genes Dev..

[118] Romanova L., Kellner S., Katoku-Kikyo N., Kikyo N. (2009). Novel role of nucleostemin in the maintenance of nucleolar architecture and integrity of small nucleolar ribonucleoproteins and the telomerase complex. J. Biol. Chem..

[119] Romanova L., Grand A., Zhang L., Romanova L., Grand A., Zhang L., Rayner S., Katoku-Kikyo N., Kellner S., Kikyo N. (2009). Critical role of nucleostemin in pre-rRNA processing. J. Biol. Chem..

[120] Hsu J.K., Lin T., Tsai R.Y.L. (2012). Nucleostemin prevents telomere damage by promoting PML-IV recruitment to SUMOylated TRF1. J. Cell Biol..

[121] Seyed-Gogani N., Rahmati M., Zarghami N., Asvadi-Kermani I., Hoseinpour-Feyzi M.A., Moosavi M.A. (2014). Nucleostemin depletion induces post-g1 arrest apoptosis in chronic myelogenous leukemia k562 cells. Adv. Pharm. Bull..

[122] Maki N., Takechi K., Sano S., Tarui H., Sasai Y., Agata K. (2007). Rapid accumulation of nucleostemin in nucleolus during newt regeneration. Dev. Dyn..

[123] Kaneko J., Chiba C. (2009). Immunohistochemical analysis of Musashi-1 expression during retinal regeneration of adult newt. Neurosci. Lett..

